# Does the *melatonin receptor 1B* gene polymorphism have a role in postoperative delirium?

**DOI:** 10.1371/journal.pone.0207941

**Published:** 2018-11-27

**Authors:** Elizabeth Mahanna-Gabrielli, Todd A. Miano, John G. Augoustides, Cecilia Kim, Joseph E. Bavaria, W. Andrew Kofke

**Affiliations:** 1 Department of Anesthesiology and Critical Care, University of Pennsylvania Perelman School of Medicine, Philadelphia, Pennsylvania, United States of America; 2 University of Pennsylvania Perelman School of Medicine, Philadelphia, Pennsylvania, United States of America; 3 The Center for Applied Genomics, Children’s Hospital of Philadelphia, Philadelphia, Pennsylvania, United States of America; 4 Department of Surgery, University of Pennsylvania Perelman School of Medicine, Philadelphia, Pennsylvania, United States of America; Beth Israel Deaconess Medical Center, UNITED STATES

## Abstract

**Introduction:**

Patients undergoing cardiac surgery are at high risk for postoperative delirium, which is associated with longer hospital and intensive care lengths of stays, increased morbidity and mortality. Because sleep disturbances are common in delirium, melatonin has been an area of interest in the treatment of delirium. The rs10830963 single nucleotide polymorphism of the *melatonin receptor 1B* gene can cause pathological dysfunction of this receptor and is associated with delayed morning offset of melatonin. We hypothesized patients undergoing aortic cardiac surgery who have the risk genotype of a *melatonin receptor 1B* polymorphism would have a higher incidence of postoperative delirium.

**Methods:**

Ninety-eight patients undergoing aortic root or valve surgery underwent analysis for *melatonin receptor 1B* single nucleotide polymorphism, rs10830963. Using a validated method, CHART-DEL, all charts were retrospectively reviewed and scored for the presence of delirium while blinded to the results of the *melatonin receptor 1B* gene polymorphism.

**Results:**

Genotyping for *melatonin receptor 1B* polymorphism was acceptable in 76 subjects of European descent of which 18 (23.7%) had delirium. Four of seven subjects with the risk genotype had delirium versus only 20.3% of subjects without the risk genotype. This carried an odds ratio of 5.2 (1.0, 26.1), p = 0.050.

**Conclusion:**

This observation suggests a role of the risk genotype of a *melatonin receptor 1B* polymorphism in the development of postoperative delirium. These hypotheses generating results warrant further prospective studies in a larger cohort group with delirium, circadian rhythm and melatonin assessments.

## Introduction

Delirium is an acute disturbance in attention, awareness and cognition that tends to fluctuate overtime and is not better explained by another neurocognitive disorder. [1, 2] Postoperative delirium is the most common neuropsychiatric complication post cardiac surgery, ranging between 21–54%. [3–5] It is associated with longer intensive care unit (**ICU**) and hospital length of stay and increased postoperative morbidity and mortality. [6, 7] Patients who experience postoperative delirium have increased readmission rates, cognitive dysfunction, poor health-related quality of life and increased mortality. [8, 9] Given the incidence and associated morbidity, delirium is an important complication after cardiac surgery.

Melatonin is a neurohormone secreted by the retina and pineal gland during dark hours and it regulates the circadian rhythm. This regulation occurs via the receptors in the suprachiasmatic nucleus. At high levels it has hypnotic effects and influences the onset, maintenance and length of sleep. [10] Because sleep and circadian rhythm disturbances affect over 75% of patients with delirium, melatonin has been an area of interest in the treatment of delirium. [11] However, whether altered melatonin metabolism leading to circadian disruption causes delirium is unknown.

In addition, dysfunctional melatonin receptors result in altered melatonin signaling. Two membrane melatonin receptors, MT1 and MT2, and nuclear receptors have been identified and found to be active in humans. Multiple studies have investigated the *melatonin receptor 1B* gene, ***MTNR1B***, polymorphisms. The minor allele, G, of the single nucleotide polymorphism (**SNP**) rs10830963, occurs in 30% of people of European descent and has been associated with altered melatonin signaling leading to multiple pathologies. [12–18] One prior prospective, cohort study has investigated the relationship between the risk genotype and delirium. However, the analysis yielded 93% of subjects having the minor allele calling into question the methods or generalizability of that sample population. [19]

We hypothesized patients undergoing aortic cardiac surgery who have the GG genotype for rs10830963 would have a significantly higher incidence of postoperative delirium as a result of altered melatonin signaling.

## Methods

We performed a retrospective cohort study of 98 patients prospectively enrolled in an observational study on adult patients undergoing non-emergent cardiac surgery: deep hypothermic cardiac arrest aortic surgery or aortic valve replacement surgery. [20] The protocol was approved by the institutional biomedical review board and subjects provided written informed consent for the study including having their medical records used in research. Subjects with genotyping for the SNP, rs10830963, of *MTNR1B* were included. Subjects of non-European descent were only included in a sensitivity analysis, due to wider variance of minor alleles across populations, concern for non-transferability of loci across human populations and the potential for population stratification bias. [21, 22] These biases could lead to increased type-I error.

### Surgery and anesthesia

The deep hypothermic cardiac arrest protocol used at University of Pennsylvania has been extensively described. [23–25] Briefly, patients undergo balanced general endotracheal anesthesia with direct intra-arterial blood pressure monitoring and cardiac output monitoring via an oximetric pulmonary arterial catheter (Baxter/Edwards, Deerfield, IL). Temperature is continuously measured in the nasopharynx and bladder. For deep hypothermic cardiac arrest patients, retrograde cerebral perfusion is initiated via superior vena cava cannula with its tip cephalad to the azygos vein and continued for the duration of deep hypothermic cardiac arrest at 10°C with perfusion pressure 25 mmHg, flow 200–300 mL/min and 10° Trendelenberg position. Deep hypothermic cardiac arrest is less than one hour, after which withdrawal of cardiopulmonary bypass is affected. Aortic valve replacement patients were similarly managed with hypothermic cardiopulmonary bypass but without retrograde cerebral perfusion.

### Single nucleotide polymorphism genotyping methods

SNP genotyping has become an established method for generating genetic profiles using custom targeted SNP assays based on the Illumina GoldenGate system. The GoldenGate custom multiplex platform genotypes between 96 and 1536 SNPs (in increments of 96) using primer extension and ligation reactions. [26, 27] The custom GoldenGate reagent kit included the rs10830963 SNP. Call rates are typically over 99%, with very high reproducibility and accuracy.

### Delirium assessment

A validated algorithm for medical record-based delirium assessment, CHART-DEL, was used for the determination of the presence of postoperative delirium. This method has been validated with a sensitivity of 74% and specificity of 83%, and overall agreement between chart and interviewer ratings is 82%. [28, 29] The Hospital Elder Life Program, which distributes the CHART-DEL protocol, includes training materials. Training was completed prior to starting the chart review. [30] Chart review was blinded to the results of the MTNR1B gene polymorphism. Per CHART-DEL method, EMG, a neurointensivist with formal National Institute of Aging sponsored training in delirium, reviewed the entire medical record of each subject and recorded all incidences of confusion, altered thinking, or altered mental state documented by attending physicians, house staff, bedside nurses and other health care providers. [28, 29] As per DSM-V criteria, each incidence of confusion cannot be better explained by another acute or chronic neurocognitive dysfunction. [2] Special attention was payed to key trigger words and phrases identified as highly associated with delirium. [29, 30] The source of the information, approximate time of onset and total duration was recorded. Evidence of agitation and reversibility or improvement was also recorded. Using CHART-DEL, it is possible to categorize the diagnosis into “Definite,” “Probable,” “Possible,” “Uncertain,” or “No evidence.” “Definite” requires the diagnosis to be unequivocal with an experienced reference standard rater such as an attending neurologist, geriatrician, psychiatrist or intensivist having made and recorded the diagnosis of delirium. “Probable” requires all Confusion Assessment Method features to be present in the notes: acute onset/fluctuation, inattention, disorganized thinking, and altered level of consciousness. [1] “Possible” is used if not all the Confusion Assessment Method features were present, but at least 2 or more plus other supporting features. “Uncertain” is used in cases when a brief mention of confusion is made but no further description or details of the episode and no further comment by any other provider. “No evidence” requires no evidence of any confusion during the hospital stay. It was decided a priori delirium would be determined as present in patients rated as “definite” and “probable.”

### Cognitive assessment

The Mini-Mental State Examination, 2^nd^ Edition brief version (MMSE-2 BV) was prospectively given preoperatively upon admission to screen for pre-existing cognitive impairment. The MMSE-2 BV is used as a rapid cognitive screener when not in the context of referral for a specific cognitive impairment. It is scored over a range of 0 to 16 points. The specific tasks test orientation, registration and recall. A cut off score of less than 14 has a sensitivity of 80% and specificity of 81% in discriminating dementia from normal cognitive function. [31]

### Data analysis

Baseline characteristics are described using descriptive statistics. Continuous variables are presented as median with interquartile range and were analyzed using the Wilcoxon rank sum test. Categorical data are presented as counts with percentages, and were analyzed using Fisher’s exact test. The primary outcome was analyzed using Fisher’s exact test in unadjusted analysis, and with logistic regression in adjusted analysis on subjects of European descent. We a priori decided to adjust for baseline cognitive function (measured using the cut off score of less than 14 on the MMSE-2 BV) and age. Subsequent variables that were found to have a relative imbalance between the groups were also controlled for in additional models. We included a maximum of two variables in all logistic regression to limit over-fitting. [32, 33]

## Results

Eighty of the enrolled subjects had acceptable call scores for the rs10830963 SNP of *MTNR1B*. Eighteen of the enrolled subjects did not have acceptable samples for determination of the rs10830963 polymorphism and were removed from the cohort. Seventy-six patients were of European descent, 2 African-American, 1 Hispanic and 1 of Asian descent. Only the 76 subjects of European descent were included in the primary analysis. Fifteen underwent deep hypothermic cardiac arrest. Among the enrolled subjects the median call score for all SNPs evaluated was 1.0 with IQR 0.989–1.0 and range 0.213–1.0; and mean call score was 0.926 +/ 0.194. Seven subjects had the risk genotype, GG. There was no significant difference in age, sex, education, steroid use, history of stroke or stroke risk factors between the patients with and without the risk genotype. Subjects with the risk genotype had a significantly shorter duration of anesthesia. Table 1

**Table 1:** Baseline characteristics in patients with the risk genotype or nonrisk genotype.

Characteristic	rs10830963 GG, n = 7	rs10830963 non-GG, n = 69	p-value
Age, years, med (IQR)	77 (70–79)	67 (56–76)	0.24
Male, n (%)	7 (100.0)	53 (76.8)	0.33
Body Mass Index, med (IQR)	27.3 (25.9–29.3)	27.5 (25.5–30.4)	0.75
MMSE-2 BV <14, n (%)	1 (14.3)	6 (8.7)	0.50
Education level <12 years, n (%)	5 (71.4)	46 (66.7)	1.00
Deep Hypothermic Cardiac Arrest, n(%)	2 (28.6)	13 (18.8)	0.62
Anesthesia duration, hours, med (IQR)	5.8 (4.9–5.9)	6.9 (5.6–8.4)	0.03
Total comorbidities, med (IQR)	3 (1–4)	3 (2–4)	0.45
Substance abuse, n (%)	0 (0.0)	3 (4.1)	1.00
Steroid, n (%)	3 (42.9)	22 (31.9)	0.68
Stroke, n (%)	1 (14.3)	4 (5.8)	0.39
Transient ischemic attack, n (%)	1 (14.3)	3 (4.4)	0.33
Carotid endarterectomy, n (%)	0 (0.0)	2 (2.9)	1.00
Hypertension, n (%)	5 (71.4)	48 (69.6)	1.00
Diabetes mellitus, n (%)			0.38
- None	5 (71.4)	57 (82.6)	
- Non-Insulin Dependent	1 (14.3)	8 (11.6)	
- Insulin Dependent	1 (14.3)	4 (5.8)	
Right carotid artery stenosis, n (%)			0.72
- None	5 (71.4)	43 (76.8)	
- < 50%	2 (28.6)	13 (18.8)	
- ≥ 50%	0 (0.0)	3 (4.4)	
Left carotid artery stenosis, n (%)			0.73
- None	5 (71.4)	52 (75.4)	
- < 50%	2 (28.6)	14 (20.3)	
- ≥ 50%	0 (0.0)	3 (4.4)	

IQR: interquartile range; MMSE-2 BV: Mini-Mental State Examination 2^nd^ Edition brief version, score range 0–16

Delirium was present in 18 subjects (23.7%). Of these subjects, nine had a definitive diagnosis of delirium recorded in the chart, and nine had all features of the Confusion Assessment Method consistent with “probable” delirium. [1] Delirium was not present in 58 subjects with 52 rated as “no evidence” of delirium, 5 rated as “uncertain”, and 1 subject rated as “possible” delirium. Of the subjects with the risk genotype, 2 had “definite” delirium, 2 had all features of the Confusion Assessment Method and “probable” delirium, and 3 with “no evidence” of delirium. Four of seven patients with the risk genotype had delirium versus 20.3% (14/69) without the genotype. Baseline characteristics stratified by delirium is shown in Table 2. Subjects with delirium trended to older age.

**Table 2:** Baseline characteristics stratified by delirium status.

Characteristic	Delirium n = 18	No Delirium n = 58	p-value
Age, years, med (IQR)	74 (69–79)	66 (54–76)	0.08
Male, n (%)	13 (72.2)	47 (81.0)	0.51
Body Mass Index, med (IQR)	29.2 (25.9–31.4)	27.1 (25.5–30.4)	0.31
MMSE-2 BV <14, n (%)	2 (11.1)	5 (8.6)	0.67
Education level <12 years, n (%)	12 (66.7)	39 (67.2)	1.00
Deep Hypothermic Cardiac Arrest, n(%)	3 (16.7)	12 (20.7)	1.00
Anesthesia duration, hours, med (IQR)	5.9 (5.1–8.4)	6.7 (5.7–7.9)	0.23
Total comorbidities, med (IQR)	4 (3–5)	3 (2–4)	0.13
Substance abuse, n (%)	0 (0.0)	3 (5.2)	1.00
Steroid, n (%)	5 (27.8)	20 (34.5)	0.77
Stroke, n (%)	0 (0.0)	5 (8.6)	0.33
Transient ischemic attack, n (%)	2 (11.1)	2 (3.5)	0.24
Carotid endarterectomy, n (%)	1 (5.6)	1 (1.7)	0.42
Hypertension, n (%)	15 (83.3)	38 (65.5)	0.24
Diabetes mellitus, n (%)			0.38
- None	13 (72.2)	49 (84.5)	
- Non-Insulin Dependent	3 (16.7)	6 (10.3)	
- Insulin Dependent	2 (11.1)	3 (5.2)	
Right carotid artery stenosis, n (%)			0.42
- None	16 (88.9)	42 (72.4)	
- < 50%	2 (11.1)	13 (22.4)	
- ≥ 50%	0 (0.0)	3 (5.2)	
Left carotid artery stenosis, n (%)			0.42
- None	15 (83.3)	42 (72.4)	
- < 50%	2 (11.1)	14 (24.1)	
- ≥ 50%	1 (5.6)	2 (3.5)	

IQR: interquartile range; BMI: Body Mass Index; MMSE-2 BV: Mini-Mental State Examination 2^nd^ Edition brief version, score range 0–16

The risk genotype, GG, was found to have an odds ratio (OR) of 5.2, 95% confidence interval (CI) [1.0, 26.1], p = 0.050 for postoperative delirium in the primary unadjusted analysis Table 3. Subsequent logistic regression analysis controlling for pre-existing dementia, as defined by MMSE-BV 2 score less than 14, showed the risk genotype had an OR 5.2 [1.0, 25.9] for postoperative delirium. When controlling for age the risk genotype had an OR 4.7 [0.9, 24.1] for postoperative delirium. The sensitivity analysis included 2 African American,1 Hispanic and 1 Asian patient is shown in Table 4.

**Table 3:** Primary outcome of delirium by genotype.

	rs10830963 GG, n = 7	rs10830963 non-GG, n = 69	Odds Ratio (95%CI)
Unadjusted	4 (57.1)	14 (20.3)	5.2 (1.0, 26.1), p = 0.050^a^
Adjusted for dementia			5.2 (1.0, 25.9), p = 0.045^b^
Adjusted for age			4.7 (0.9, 24.1), p = 0.064^b^
Adjusted for anesthesia duration			5.0 (0.94–26.6), p = 0.058^b^
Adjusted for stroke / TIA			5.2 (1.0–26.2), p = 0.044^b^
Adjusted for sex			6.5 (1.2–34.3), p = 0.027^b^

^a-^Fisher’s exact test;

^b-^ logistic regression model;

TIA: transient ischemic attack

**Table 4:** Sensitivity analysis with all races included^a^.

	rs10830963 GG, n = 7	rs10830963 non-GG, n = 73	Odds Ratio (95%CI)
Unadjusted	4 (57.1)	14 (19.2)	5.6 (1.1, 28.0), p = 0.042^b^
Adjusted for dementia			5.5 (1.1, 27.7), p = 0.037^c^
Adjusted for age			4.9 (0.9, 25.6), p = 0.055^c^
Adjusted for anesthesia duration			5.3 (1.0–28.3), p = 0.049^c^
Adjusted for stroke / TIA			5.6 (1.1–27.9), p = 0.036^c^
Adjusted for sex			7.1 (1.4–37.3), p = 0.020^c^

^a-^ Include 4 additional patients (African-American n = 2, Hispanic n = 1, and Asian n = 2);

^b-^ Fisher’s exact test;

^c-^ logistic regression model;

^d-^ TIA: transient ischemic attack

## Discussion

Delirium after cardiac surgery is a common and morbid complication. Unfortunately, the exact pathophysiological mechanism is unknown, although inflammation, altered circadian rhythms and sleep disturbances are highly associated with postoperative delirium. [34] We hypothesized the risk genotype, GG, for rs10830963 of *MTNR1B* gene would be associated with postoperative delirium after cardiac surgery. Via chart review, we determined 23.7% of subjects had delirium, which is in agreement with previously reported rates in post cardiac surgery patients. [3–5, 9] In our cohort only 7 subjects were found to have the risk genotype, and our results should therefore be considered hypothesis generating. We found the risk genotype had a strong but imprecise association with delirium with an OR 5.2 [1.0, 26.1]. In analyses that adjusted for pre-existing dementia, age, anesthesia duration, sex and history of stroke or transient ischemic attack, the point estimates were similar, again with wide confidence intervals. In addition, a subsequent sensitivity analysis with all races in the cohort had similar point estimates and confidence intervals. Given the retrospective nature of our assessment, it is possible we misclassified subjects as having or not having delirium. However, any misclassification of delirium diagnosis should be non-differential across genotype groups, and any bias would be expected to be towards the null. [35]

Melatonin signaling affects two key processes: the circadian rhythm and inflammatory pathways. Both may influence the development of postoperative delirium. Melatonin is a key regulator of the circadian rhythm via its interaction with the master circadian pacemaker, the suprachiasmatic nucleus. In cardiothoracic surgery patients, sleep deprivation has been found to precede postoperative delirium and a reduction in serum melatonin and abnormal circadian rhythms have been associated with postoperative delirium and age. [34, 36] Melatonin is also intimately involved in up and down regulation of the immune system and inflammatory processes. [37] Aberrancy in melatonin signaling and levels has been shown in septic patients. In one prospective, controlled study, septic patients in the ICU were found to have nearly abolished circadian rhythm with significantly reduced amplitude with no regular daytime decline of a urinary melatonin metabolite in contrast to non-septic ICU patients and control patients. [38] A key hypothesis of the pathophysiology of postoperative delirium is systemic inflammation leading to subsequent neuroinflammation and neurotransmitter dysfunction. [39–41]

There are 2 alleles of rs10830963 SNP of *MTNR1B* found in humans: C and G. These genetic variances have been extensively studied. The G allele has been found to be a risk allele and genotype GG has repeatedly been shown to be pathologic. [12, 14] One recent prospective, cohort study has investigated rs10830963 polymorphism’s effect on sleep status, circadian rhythm and melatonin traits. [42] The GG genotype was significantly correlated with a longer duration of melatonin levels, delayed decrease in melatonin after light exposure and forced early awakening led to increased pathology. [13, 42]

Our preliminary retrospective study was not designed to elicit the pathophysiology of how the risk genotype of rs10830963 of the *MTNR1B* gene incurs an increased risk of postoperative delirium. However, it has repeatedly been shown this genotype is associated with altered melatonin signaling. One could hypothesize possible explanations. First, often in the hospital, especially on post-surgical wards, patients are awoken early as the team rounds. This forceful early wakening in the risk genotype patients would occur when the melatonin levels are still elevated. This may lead to further disruption in the circadian rhythm placing the patient at increased risk for sleep disturbances and delirium. In addition, this SNP may affect other interventions used to treat and prevent delirium including sleep hygiene protocols and medications to promote sleep. Future prospective studies with a larger cohort, prospective delirium testing, melatonin level measurements and circadian rhythm assessments are needed to further elucidate the relationship between the risk genotype and delirium.

Determining if the GG allele does indeed incur an increased risk of delirium could be an important step forward in precision medicine. Exogenous administration of melatonin or ramelteon, a synthetic melatonin agonist, has been investigated as a preventative therapy for delirium. However, trials investigating this have shown inconclusive results. [43–45] The complex diurnal regulation of melatonin and its effect on its multitude of receptors and inflammation feedback loops, may lead to unforeseen effects of exogenous administration. Specifically, inappropriately high melatonin levels may have deleterious effects such as hypoactive delirium. [34, 42] The lack of discrimination for who receives exogenous melatonin without regard to the patient’s endogenous production or *MTNR1B* genotype may play a role in the mixed results of the trials. Genotyping may yield information regarding patients in whom melatonin-directed therapy may be more effective.

## Conclusion

Our observation suggests a role of the risk genotype, GG, of rs10830963 SNP of *melatonin receptor 1B* in the development of postoperative delirium. These hypotheses generating results warrant further prospective studies in a larger cohort group with delirium, circadian rhythm and melatonin assessments.
